# Immunohistochemical Study of p53 and Ki-67 in Benign and Malignant Prostatic Lesions

**DOI:** 10.7759/cureus.113305

**Published:** 2026-07-24

**Authors:** Ayushi Shrivastava, Vijayalaxmi Patil, Santosh Patil

**Affiliations:** 1 Pathology, BLDE (Deemed to be University) Shri BM Patil Medical College, Hospital and Research Centre, Vijayapura, IND; 2 Urology, BLDE (Deemed to be University) Shri BM Patil Medical College, Hospital and Research Centre, Vijayapura, IND

**Keywords:** benign prostate, immunohistochemistry(ihc), ki67, p53 expression, prostate adenocarcinoma

## Abstract

Introduction

Prostate adenocarcinoma is a significant health concern with variable clinical outcomes. While markers such as serum prostate-specific antigen (PSA) are used for screening, these have limitations in predicting tumor behavior. The identification of reliable prognostic biomarkers is crucial for guiding treatment decisions. This study aimed to evaluate the immunohistochemical expression of p53 and Ki-67 in prostatic lesions and correlate their expression with Gleason grade.

Methodology

This cross-sectional study was conducted on 50 prostate specimens, comprising 25 cases of Prostate adenocarcinoma and 25 cases of benign prostatic hyperplasia (BPH). Immunohistochemical (IHC) staining for p53 and Ki-67 was performed on all cases. The expression of these markers was then correlated with the histological grade (using the Gleason grading system) and preoperative serum PSA levels.

Results

Patients with prostate adenocarcinoma presented at a slightly older age than those with BPH. Serum PSA levels showed wide variability among patients with carcinoma and did not correlate significantly with Gleason grade. While p53 expression was observed in a subset of Prostate adenocarcinomas, p53 is expressed but lacks significant clinicopathological correlation. In contrast, the Ki-67 proliferation index was significantly higher in prostate adenocarcinoma than in BPH (p<0.05) and demonstrated a significant positive correlation with increasing Gleason scores, indicating its value as a marker of tumor proliferation, poor differentiation, and aggressive biological behavior.

Conclusion

The present study demonstrates that the expression of Ki-67, a marker of cellular proliferation, is significantly increased in malignant lesions when compared with benign lesions. Tumors showing positivity for p53 and Ki-67 on biopsy are likely to exhibit aggressive biological behavior and may therefore serve as independent prognostic indicators in cases with a higher risk of metastasis. However, further prospective studies incorporating long-term clinical follow-up and detailed molecular genetic analyses are required to elucidate the biological role of these immunohistochemical markers and to determine their true prognostic value in patients with prostate adenocarcinoma.

## Introduction

Prostate adenocarcinoma ranks as the second most common cancer diagnosed in males worldwide and a leading cause of cancer-related mortality [1]. Its incidence escalates sharply with age, remaining rare before 40 years but surging particularly after the seventh decade [2]. Early-stage disease exhibits heterogeneous behavior, with many tumors remaining indolent over years, while others progress aggressively, leading to death within a few years. In contrast, benign prostatic hyperplasia (BPH) affects 20% of men at the age of 40 years and nearly 90% by the eighth decade, causing substantial urological morbidity via lower urinary tract symptoms but lacking malignant potential [3]. Both conditions predominantly afflict elderly men, yet prostate adenocarcinoma differs markedly from BPH in its morbidity. Pathogenesis involves hormonal, genetic, and environmental factors, culminating in sequential genetic alterations, including early inactivation of tumor-suppressor genes [4].

Diagnosis integrates digital rectal examination, serum prostate-specific antigen (PSA) testing, multiparametric MRI, and biopsy [5]. Grading is performed using the modified Gleason system and World Health Organization (WHO) Prognostic Grade Groups. Grade Group 1 (≤6) indicates low-risk; Group 2 (3+4=7) and Group 3 (7) represent intermediate-risk; and Group 4 (8) and Group 5 (9-10) signify high-risk, poorly differentiated tumors [6]. The molecular pathogenesis of prostate adenocarcinoma centrally involves dysregulation of the PI3K/AKT/mTOR pathway, occurring in 30-50% of cases. Normally, receptor tyrosine kinases activate PI3K to generate PIP3, which recruits and activates Akt to promote cell growth and survival [7]. The tumor suppressor PTEN negatively regulates this pathway by dephosphorylating PIP3. PTEN loss - through deletion, mutation, or epigenetic silencing - causes Akt hyperactivation, driving uncontrolled proliferation [8]. Additionally, inactivation of the TP53 tumor suppressor, through mutation or deletion, contributes to genomic instability and resistance to apoptosis [9]. This pathway dysregulation, alongside *ETS* gene fusions, facilitates tumor progression and the development of castration-resistant prostate adenocarcinoma.

Prostate adenocarcinoma has a relatively low frequency of *TP53* mutations, which are of two types: wild-type p53 protein that shows weak, focal, or heterogeneous nuclear staining because of transient physiological stabilization in response to cellular stress, and mutant p53 protein that commonly demonstrates strong, diffuse nuclear accumulation owing to its prolonged half-life. Conversely, certain *TP53* mutations may result in complete absence of p53 staining (null pattern), but this rate increases substantially in castrate-resistant, locally advanced, and metastatic tumors. Loss of p53 is associated with higher tumor grade, advanced stage, increased lymph node metastasis, and poorer response to therapy. Immunohistochemical studies demonstrate that abnormal p53 expression due to accumulation of dysfunctional protein and correlates with aggressive clinical behavior, lymphovascular invasion, and extra-prostatic spread [10]. Ki-67 expression rises progressively from normal prostate through premalignancy to carcinoma, exceeding that in BPH, while p53 overexpression correlates with advancing stage and Gleason score [11].

Ki-67 and p53 show promise but yield inconsistent independent prognostic value in prostate adenocarcinoma. Therefore, this study evaluates immunohistochemical expression of p53 and Ki-67 in BPH and prostate adenocarcinoma, correlating findings with Gleason score and serum PSA levels.

## Materials and methods

Study design and sample size

This was a hospital-based cross-sectional observational study including 25 patients per group (i.e., a total sample size of 50, assuming equal group sizes of prostate adenocarcinoma and BPH cases).

Ethical approval

Ethical approval for the study was obtained from the Shri BM Patil Medical College, Hospital and Research Centre, Vijayapura Institutional Ethics Committee (reference number: BLDE(DU)IEC-SBMPMC/124/2023-24).

Inclusion and exclusion criteria

Histologically diagnosed cases of prostate adenocarcinoma and BPH were included in the study, and metastatic carcinomas of the prostate were excluded.

Data collection

Specimens of the prostate, such as prostatic needle biopsy (10 samples) and TURP CHIPS (chips of transurethral resection of the prostate, 15 samples), were received in the Histopathology Section, Department of Pathology, BLDE (Deemed to be University), Shri BM Patil Medical College, Hospital and Research Centre, Vijayapura, Karnataka. Information regarding Clinical details, PSA levels, and findings on imaging, if any, was obtained. The tissue was preserved in 10% buffered formalin and processed routinely. Three micro-thick sections were prepared from each tissue block. One section was stained with hematoxylin and eosin (H&E) for morphologic diagnosis, and Gleason’s score and grade, and the other two sections were mounted on poly-L-lysine-coated slides, which were used for p53 and Ki-67 immunohistochemical staining.

Immunohistochemical analysis

Positive and negative controls were processed at the same time. Strong brown staining in cell nuclei indicated positive results. Staining was quantified by the percentage of tumor cells reacting to the antibody. Slides were first scanned at ×40 magnification to locate regions with the highest positive cell density. These areas were then assessed at ×400 magnification, where the proportion of positive cells to total cells was determined. At least 500 cells were counted per slide, including only those positive for the target marker.

Statistical analysis

The data obtained were entered into a Microsoft Excel (Redmond, Washington, USA) sheet, and statistical analysis was performed using the Statistical Package for Social Sciences (version 20). Results are presented as mean ± standard deviation (SD), counts and percentages, and diagrams. Normally distributed continuous variables between two groups were compared using an independent t-test, and the Mann-Whitney U test was used for not normally distributed variables. Categorical variables between two groups were compared using a chi-square test. A p<0.05 was considered statistically significant. All statistical tests were two-tailed.

## Results

The study was equally divided into two groups, consisting of 25 cases of BPH and 25 cases of prostate adenocarcinoma. Patients with prostate adenocarcinoma presented at an older age compared to those with BPH. The age of patients with BPH ranged from 40 to 98 years, with a mean age of 69 years, whereas the age of patients with prostate adenocarcinoma ranged from 52 to 91 years, with a mean age of 71.5 years. Serum PSA levels in prostate adenocarcinoma showed a wide variation, ranging from a minimum of 10.3 ng/mL to a maximum of 96.4 ng/mL (reference range of serum PSA used was from 10 ng/mL to 100 ng/mL).

The distribution of Gleason scores among cases of prostate adenocarcinoma (n=25) demonstrated marked heterogeneity, with a predominance of Gleason score 7 observed in 12 cases (48%). Within this group, the most frequent pattern was 4+3, seen in seven cases, followed by the 3+4 pattern in five cases. Gleason score 8 was identified in six cases (24%), comprising three cases with a 3+5 pattern, two cases with a 4+4 pattern, and one case with a 5+3 pattern. Gleason score 9 was noted in four cases (16%), with equal distribution between the 4+5 and 5+4 patterns, with two cases each. Gleason score 10 was observed in two cases (8%), both exhibiting a 5+5 pattern. A single case (4%) demonstrated Gleason score 6 with a 3+3 pattern.

The analysis of the relationship between serum PSA levels and Gleason grade group revealed no statistically significant association. The distribution of PSA categories across grade groups was variable without a clear linear progression; for instance, while the highest PSA level (80.1+) was present in both Grade group 2 and Grade group 4 tumors, it was absent in Grade group 5.

Distribution of p53 expression in prostate adenocarcinoma and BPH

**Figure 1:**
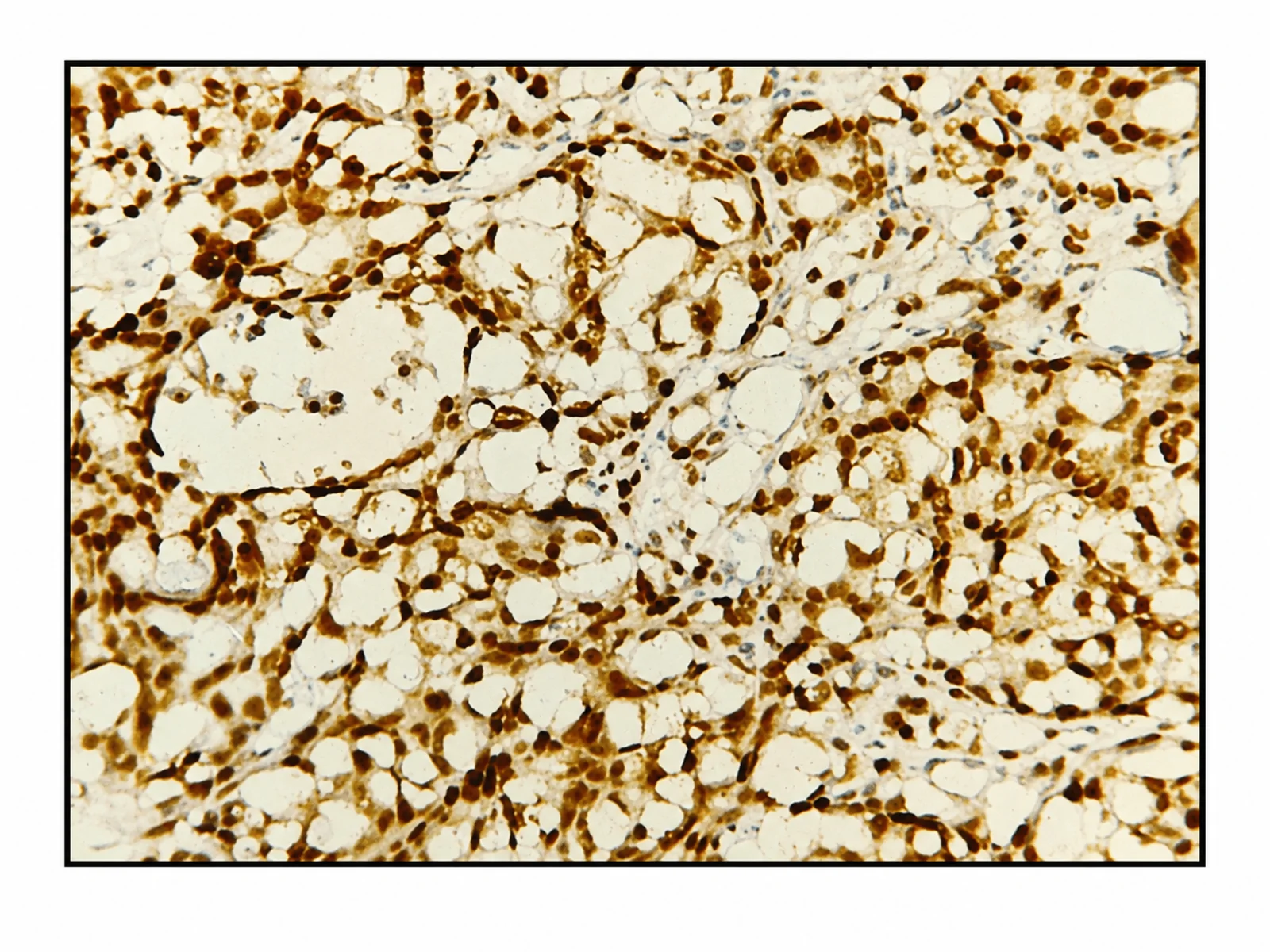
Photomicrograph showing high expression of p53 nuclear positivity in prostate adenocarcinoma (IHC-40X) IHC: immunohistochemistry

The comparative analysis of p53 protein expression in prostate adenocarcinoma and BPH reveals a predominance of high immunoreactivity in both conditions, though with notable differences in distribution across lower categories (Table 1). In prostate adenocarcinoma (n=25), most cases (76%, n=19) exhibited strong (3+) p53 expression due to increased epithelial turnover associated with BPH and chronic low-grade inflammation rather than malignant transformation alone, followed by 20% (n=5) with moderate (2+) staining, and only a single case (4%) showing complete negativity (0%) (Figure 1). In contrast, BPH samples (n=25) demonstrated an even higher frequency of strong (3+) expression at 80% (n=20), with the remaining cases distributed between moderate 2+ expression (12%, n=3) and low 1+ expression (8%, n=2).

**Table 1:** p53 expression in prostate adenocarcinoma and benign prostate hyperplasia

p53 expression	Prostate adenocarcinoma	BPH
0 (0)	4%	0%
1+ (<10)	0%	8%
2+ (10-33)	20%	12%
3+ (>33)	76%	80%

Distribution of Ki67 proliferation index in prostate adenocarcinoma and BPH

**Figure 2:**
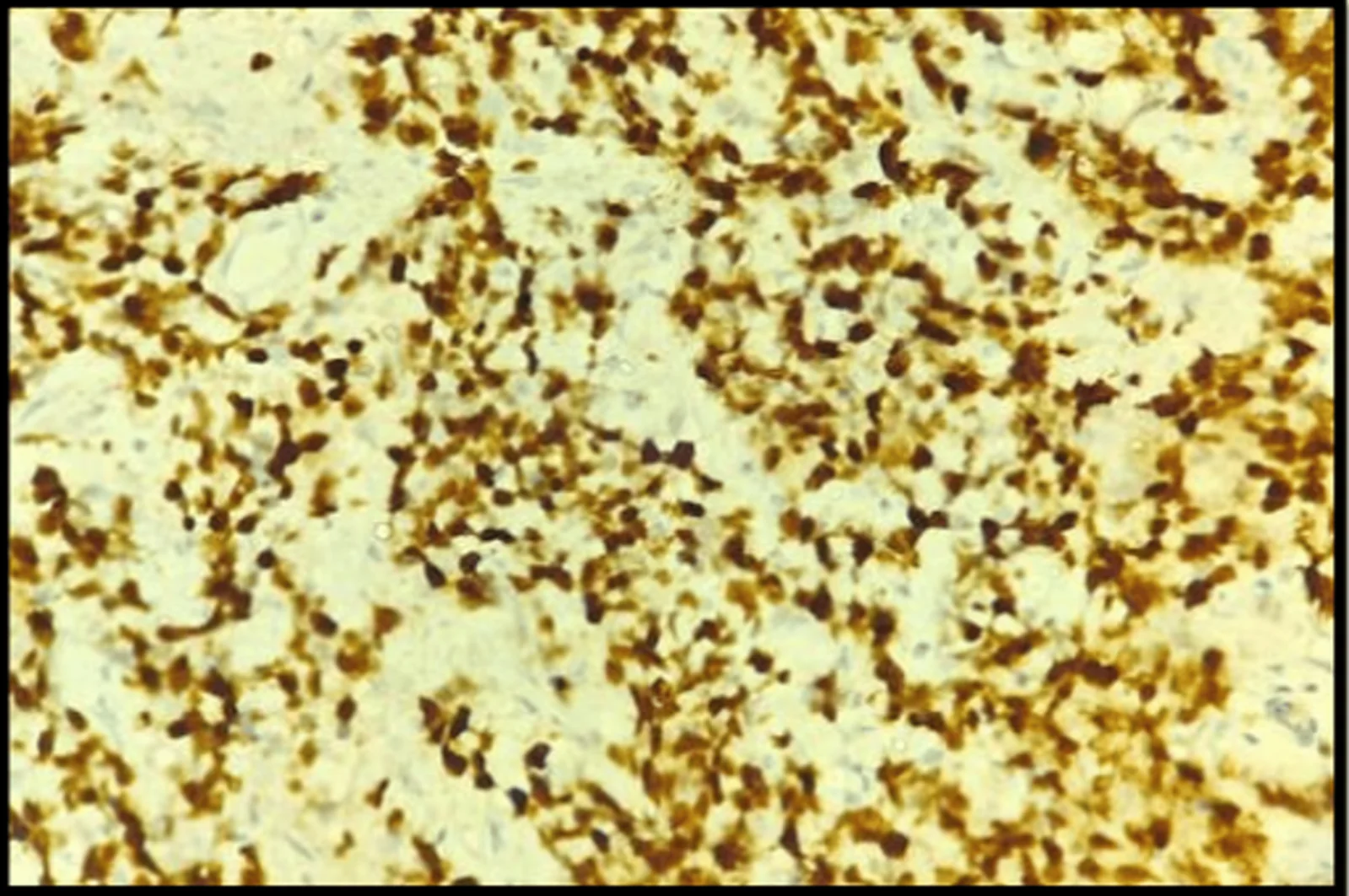
Photomicrograph showing high expression of Ki67 nuclear positivity in prostate adenocarcinoma (IHC-40X) IHC: immunohistochemistry

A comparative analysis of Ki67 proliferation index in prostate adenocarcinoma and BPH demonstrated a significant difference in proliferative activity between malignant and benign prostatic tissues (Table 2). In prostate adenocarcinoma (n=25), most cases (76%, n=19) exhibited proliferative activity, whereas BPH samples (n=25) showed almost no proliferative activity, with most cases (88%, n=22) falling into the negative category (Figure 2).

**Table 2:** Ki67 expression in prostate adenocarcinoma and benign prostate hyperplasia

Ki67 expression	Prostate adenocarcinoma	BPH
Negative (<2)	24%	88%
1+(2-25)	76%	12%
2+(26-50)	0%	0%
3+(51-75)	0%	0%
4+(76-100)	4%	0%

The mean p53 expression was slightly higher in prostate adenocarcinoma (48±21.01) compared to BPH (45.6±19.91); however, this difference was not statistically significant (Mann-Whitney U=297.50, p=0.779). However, Ki-67 expression was markedly higher in prostate adenocarcinoma (10.68±18.06) than in BPH (0.56±0.72), and this difference was found to be statistically significant (Mann-Whitney U=68.50, p=0.0001).

Association between p53 expression and Gleason grade group

An analysis of p53 expression in relation to the Gleason grade group revealed a notable, though not statistically significant, trend. Higher grade groups (Grades 3, 4, and 5) were predominantly associated with high p53 expression (3+), comprising 85.7%, 100%, and 66.7% of cases, respectively. Pearson's chi-square test found no statistically significant association between p53 expression categories and Gleason grade group (χ²=10.253, p=0.248).

Association between Ki67 proliferation index and Gleason grade group

An analysis revealed a statistically significant association between the Ki67 proliferation index and Gleason grade group. The distribution showed a general trend of increasing proliferative activity with higher grade group. Specifically, low-grade group tumor (Grades 1 and 2) was predominantly associated with lower Ki67 indices (1+), with 80% of Grade group 2 tumors falling in the lowest category (<2%). In contrast, higher-grade group tumors (Grades 3, 4, and 5) were primarily characterized by an elevated Ki67 index (2-25%), accounting for 85.7%, 100%, and 66.7% of cases, respectively.

## Discussion

The present study aimed to evaluate the clinicopathological profile of prostate adenocarcinoma and BPH, while assessing the immunohistochemical expression of p53 and Ki67 in these lesions and correlating them with tumor grade. Given the heterogeneous biological behavior of prostate adenocarcinoma and the limitations of traditional prognostic parameters, markers such as p53 and Ki67 have garnered interest as potential adjuncts for risk stratification.

In the present study, patients with prostate adenocarcinoma presented at a higher mean age (71.5 years) compared to those with BPH (69 years), with the majority (76%) aged 70 years or older. This finding aligns with that presented in the established literature, including studies by Verma et al. and Bhat et al., which identify advancing age as a predominant non-modifiable risk factor for prostatic malignancy [12,13]. The notable age overlap between patients with BPH and carcinoma observed here underscores the diagnostic challenges in elderly populations and reinforces the necessity of histopathological and immunohistochemical evaluation for accurate differentiation.

Serum PSA levels in adenocarcinoma cases exhibited a wide range (10.3-96.4 ng/mL) with no statistically significant association with Gleason grade groups. This observation supports the growing consensus that PSA alone is an unreliable predictor of tumor aggressiveness. Similar findings were reported by Verma et al., Bhat et al., and Gangwar et al., who noted wide PSA variability across grades and emphasized that high-grade tumors may lose proportional PSA production, leading to disproportionately lower serum levels despite aggressive behavior [12-14]. Consequently, PSA should be interpreted alongside histopathological parameters rather than in isolation.

Histologically, Gleason score 7 was most frequent (48%), with a predominance of the prognostically unfavorable 4+3 pattern over 3+4. High-grade tumors (Gleason score 8-10) constituted 48% of cases, and no well-differentiated tumors were identified. This distribution reflects a substantial burden of advanced disease at presentation, consistent with Indian studies by Verma et al., Bhat et al., Gangwar et al., and Rajeswari et al., and highlights the gaps in early detection practices [12-15].

p53 immunoexpression was observed in 96% of adenocarcinoma cases, with 76% showing strong (3+) positivity. However, no significant association was found between p53 expression and Gleason grade. Moreover, p53 expression did not significantly differ between adenocarcinoma and BPH, limiting its diagnostic utility. These findings concur with those of Thompson et al., Verma et al., and Bhat et al., who concluded that p53 accumulation may reflect genomic instability rather than malignant transformation specifically, and that its mutations are infrequent in primary prostate adenocarcinoma, being more common in the advanced disease [11-13].

In contrast, Ki-67 expression demonstrated significant prognostic relevance. A statistically significant positive correlation was observed between Ki-67 labeling index and Gleason grade group (p=0.047), with higher-grade tumors exhibiting increased proliferative activity. Furthermore, Ki-67 expression was markedly higher in adenocarcinoma compared to BPH (p=0.0001), supporting its diagnostic value in distinguishing malignant from benign lesions. These findings align with those of studies by Gangwar et al., Rajeswari et al., and Muñoz et al., which identified Ki-67 as a reliable indicator of tumor proliferation and aggressiveness [14-16].

Limitations

The present study is limited by its single-center design, small cohort size, and the use of a conventional (old) immunohistochemical method for evaluating p53 expression, which may affect the generalizability of the findings. Furthermore, analyses of clinicopathological subgroups were based on even smaller subcohorts, limiting statistical power and the precision of subgroup comparisons. The cross-sectional design precluded evaluation of long-term clinical outcomes, including recurrence, metastasis, and survival. In addition, molecular analysis of *TP53* mutations was not performed, limiting the correlation between p53 protein expression and underlying genetic alterations. Interobserver variability in immunohistochemical interpretation was also not assessed.

## Conclusions

p53 expression was frequently observed in prostate adenocarcinoma. It did not show a consistent association with tumor grade or malignant status. In contrast, Ki-67 expression demonstrated a significant association with tumor grade and effectively differentiated benign from malignant prostatic lesions using the histopathological and immunohistochemical methods. These findings suggest that Ki-67 may serve as a useful adjunct to routine histopathological evaluation, particularly in cases with intermediate Gleason scores.
